# Fate of MTBE and DCPD Compounds Relative to BTEX in Gasoline-Contaminated Aquifers

**DOI:** 10.1100/tsw.2002.218

**Published:** 2002-04-24

**Authors:** L. Olivella, M. Figueras, J. Fraile, M. Vilanova, A. Ginebreda, D. Barceló

**Affiliations:** Departament de Control, Àrea d’Inspecció i Control, Agència Catalana de l’Aigua, Spain; Department of Environmental Chemistry, IIQAB-CSIC, Jordi Girona 18-26, 08034 Barcelona, Spain

**Keywords:** MTBE (methyl tertiary-butyl ether), DCPD (dicyclopentadiene), BTEX (benzene-toluene-ethylbenzene-xylene), gasoline, groundwater

## Abstract

The aim of this communication is to provide preliminary results on MTBE monitoring, and at the same time to propose some new tracers of gasoline pollution in groundwater. An overview is presented on benzene-toluene-ethylbenzene-xylene (BTEX), methyl tertiary-butyl ether (MTBE), and dicyclopentadienes (DCPD) contents in gasoline formulations. Their specific fate in gasoline-contaminated aquifers are consistent with their physical-chemical properties.

## INTRODUCTION

Methyl tertiary-butyl ether (MTBE) is by far the most commonly used fuel additive. In the U.S., it has been added in gasoline formulations since the 1970s at concentrations ranging from 15 to 30%. Its frequent occurrence in shallow groundwaters led MTBE to be included in the 1998 Contaminant Candidate list (CCL), published by the EPA[1]. The introduction of MTBE in European fuels in 1988, with concentrations ranging from 1.5 to 15%, makes it necessary to also assess its environmental impact in Europe. Dicyclopentadiene (DCPD) and 8-dihydroDCPD (8-

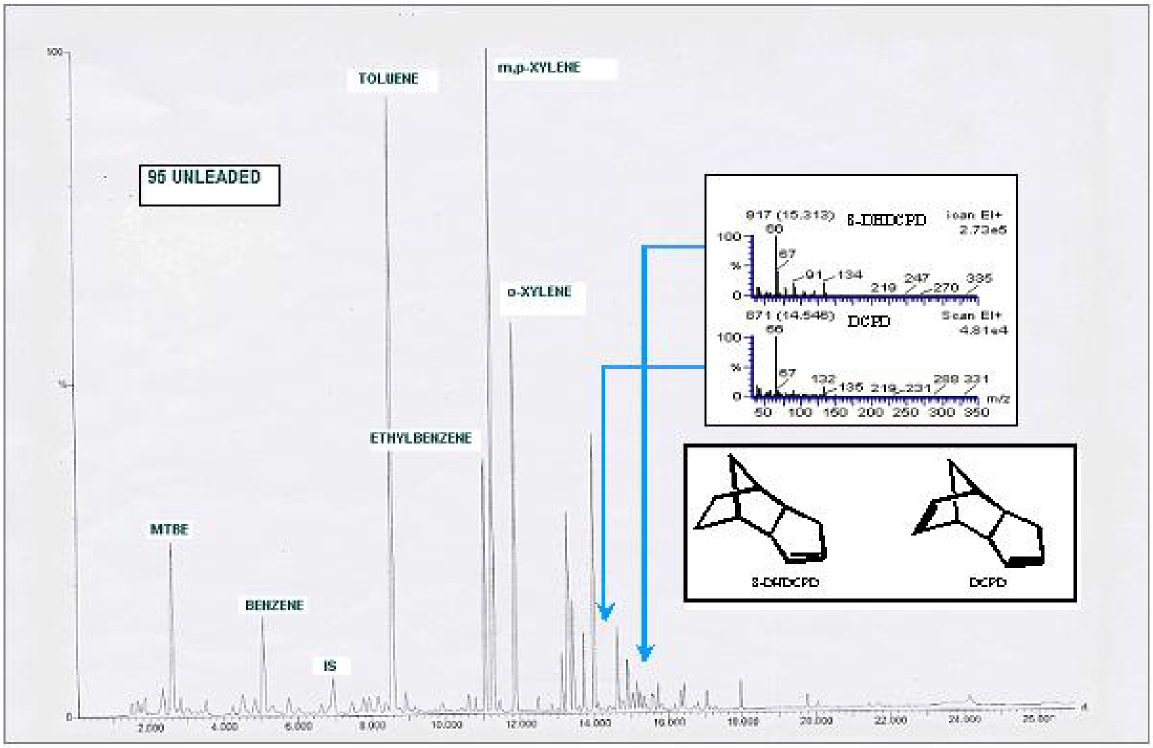

**FIGURE 1.** Chromatogram of 95–unleaded gasoline, showing the chemical structures of DCPD and 8-DHDCPD peaks and their mass spectra. (IS = Internal standard).

DHDCPD) (Fig. 1) are minor compounds in gasoline formulations, and have been previously considered responsible for odor and taste events in groundwater supplies, some years after a gasoline spill[2].

The aim of this work is to characterize gasoline formulations used in Spain, quantifying their contents of BTEX, MTBE, DCPD and 8-DHDCPD; and to monitor their relative spatial distribution in polluted groundwaters over time during a maximum of 4 years.

## GASOLINE STANDARDS AND METHODOLOGY

Two methods (Table 1) based on Headspace-GC-FID (BTEX) and purge-and-trap (MTBE-DCPDs), followed by GC-MS analysis were used in this study, according to EPA method 624[3] for water samples. Four commercial gasoline samples (Table 2) were taken from a CEPSA petrol station service between May and September 2001, and they were analyzed in less than 4 days. An initial gasoline dilution in methanol was necessary to minimize the insoluble properties of fuel in water before applying purge-and-trap methodology.

MS analysis was done in scan mode using the following ions to quantify each compound (m/c): 73 (MTBE), 78 (benzene), 91 (toluene), 105 ($C_2$-alkyl benzene), and 66 (DCPDs); α,α,α-trifluorotoluene (ion 146) was used as internal standard, at 25 ppb.

Standard of DCPD (Aldrich reagent) was obtained from AGBAR (Aguas de Barcelona, Spain), and 8-DHDCPD was quantified as DCPD, using the common ion 66 as a quantifier.

**TABLE 1**

**Summary of Analytical Conditions**

| Compounds | Rt *(min)* | Method | $R^2$ | LOD (*µg/l*) | range studied (*µg/l*) | S.D. (%) |
|---|---|---|---|---|---|---|
| MTBE | 2.56 | GC-MS | 0.9995 | 5.7 | 15,000 | 7.9 |
| Benzene | 15.81 | GC-FID | 0.9998 | 0.6 | 1,000 | 12.2 |
| Toluene | 21.02 | GC-FID | 0.9998 | 0.6 | 1,000 | 11.7 |
| Ethylbenzene | 25.42 | GC-FID | 0.9998 | 0.4 | 1,000 | 8.2 |
| *m*+*p*-Xylene | 25.75 | GC-FID | 0.9998 | 0.3 | 1,000 | 4.8 |
| *o*-Xylene | 26.98 | GC-FID | 0.9998 | 0.4 | 1,000 | 6.3 |
| DCPD | 14.49 | GC-MS | 0.9975 | 0.3 | 50 | n.q. |
| α,α,α-trifluorotoluene | 18.17 | | | | Fixed=25 ppb | |

**Note:** α,α,α-Trifluorotoluene was used as Internal standard. Abbreviations: L.O.D. = Lower detection limit; GC-MS = gas chromatography–mass spectrometry; GC-FID = gas chromatography–flame ionization detector; and n.q. = not quantified.

**TABLE 2**

**Commercial Gasoline (CEPSA) Compositions**

| | GASOLINE COMPOSITION | | (CEPSA) | |
|---|---|---|---|---|
| Date Sampling | Mai-01 | Mai-01 | Mai-01 | Sep-01 |
| | 95 Unleaded | 98 Unleaded | 97 Leaded | New 97 Unleaded |
| COMPOUNDS | (v/v) | (v/v) | (v/v) | (v/v) |
| MTBE | 4.7 | 9.1 | 5.9 | 2.1 |
| BENZENE | 0.6 | 0.6 | 0.5 | 0.6 |
| TOLUENE | 4.0 | 6.5 | 5.3 | 7.4 |
| ETILBENZENE | 1.0 | 1.7 | 1.3 | 2.2 |
| *m*+*p*-XILENE | 3.3 | 4.8 | 3.9 | 5.6 |
| *o*-XILENE | 1.1 | 1.9 | 1.4 | 2.7 |
| DCPD | 0.0022 | 0.032 | 0.0093 | 0.0050 |
| 8-DHDCPD | 0.0074 | 0.020 | 0.0046 | 0.0029 |
| DCPD/8-DHDCPD | 0.3 | 1.6 | 2.0 | 1.7 |

## TIME EVOLUTION

Three wells were monitored during a period of 4 years in order to determine possible changes in a relative concentration pattern with respect to original gasoline composition. Wells PO2372 and PO2332 are located in the neighborhood of oil refinery storage tanks in a multilayer aquifer, characterized by detritic materials (conglomerates, sands, and clays), and well PO9030 is near a petrol service station in an unconfined aquifer constituted by gravels and limestone. Results recorded in Table 3 show a rapid decay of MTBE concentrations during the first period after the spill, followed by a stabilization at concentration levels of ppb; determined mainly by its great mobility (water solubility = 50,000 mg/l) and secondly by its resistance to biodegradation. The same decreasing tendencies were found for BTEX compounds during this monitory survey. The half-lives of BTEX compounds in groundwaters are known to vary considerably from as short as 1 week to as long as 2 years[4]; this link to their slightly retarded mobility in groundwater could be related to the effects found.

**TABLE 3**

**Concentration of Gasoline Constituents in Contaminated Aquifers Over Time**

| PO2372 | ppb | | | | | | | | |
|---|---|---|---|---|---|---|---|---|---|
| | MTBE | BENZENE | TOLUENE | ETHYLBENZENE | *m+p*-XYLENE | *o*-XYLENE | DCPD | 8-DHDCPD | DCPH / 8-DHDCPD |
| nov-97 | 354 | 1016 | 14.9 | 7 | 12.2 | 22.4 | 108.0 | 125.0 | 0.9 |
| dec-98 | 333 | 1.3 | 3.6 | 7.9 | 3.5 | 9.6 | 69.2 | 92.3 | 0.7 |
| nov-99 | 11.4 | <0.6 | <0.6 | <0.5 | 1.6 | 1.1 | 45.3 | 39.9 | 1.1 |
| jan-01 | 8 | <0.6 | <0.6 | 1.2 | 2.2 | 1.2 | 54.8 | 53.8 | 1.0 |
| marc-01 | <6 | 1.6 | <0.6 | 0.7 | 1.3 | <0.5 | 67.0 | 54.0 | 1.2 |

| PO9030 | ppb | | | | | | | | |
|---|---|---|---|---|---|---|---|---|---|
| | MTBE | BENZENE | TOLUENE | ETHYLBENZENE | *m+p*-XYLENE | *o*-XYLENE | DCPD | 8-DHDCPD | DCPH / 8-DHDCPD |
| nov-99 | 10274 | 7.4 | 0.6 | <0.5 | 445 | 379 | 23.5 | 2.05 | 11.5 |
| oct-01 | 132 | 10.7 | 9.9 | 5.8 | 6.6 | 11.5 | 4.2 | 2.1 | 2 |

| PO2332 | ppb | | | | | | | | |
|---|---|---|---|---|---|---|---|---|---|
| | MTBE | BENZENE | TOLUENE | ETHYLBENZENE | *m+p*-XYLENE | *o*-XYLENE | DCPD | 8-DHDCPD | DCPH / 8-DHDCPD |
| oct-97 | 103 | 3866.0 | 610.0 | 54.0 | 180.0 | 170.0 | 28.5 | 136.0 | 0.2 |
| dec-98 | 44 | 1262.0 | 285.0 | <0.5 | 184.0 | 153.0 | 36.8 | 112.5 | 0.3 |
| nov-99 | 77 | <0.6 | <0.6 | <0.5 | 50.7 | 55.4 | 11.0 | 83.0 | 0.1 |
| jan-01 | 32 | <0.6 | 0.6 | <0.5 | 0.7 | 7.9 | 31.9 | 272.0 | 0.1 |

On the other hand, lower degradation rates of benzene and *o*-xylene found in one well (PO2372) could be tentatively explained in terms of particular redox environments[6] that allow the development of denitrifying biodegradation conditions.

Only DCPDs exhibited a high persistence, with concentrations that remained almost unchanged after 4 years; this is according to the results of biodegradation studies[7] that suggest DCPD is poorly degraded in soil and water, with estimated half-lives of 1–2 years and 4–7 years, respectively.

An overall view of these effects were clearly observed by comparing the chromatographic profiles obtained from Eurosuper gasoline pattern (Fig. 1) with those obtained from the monitory survey of well PO2372 (Figs. 2 and 3) on two different dates.

## SPATIAL DISTRIBUTION

Table 4 includes the survey well distances from the fuel spills origins, drawing pollution plumes for MTBE, which cover areas of ca. 2 km maximum, in agreement with other studies[8]. As expected from their respective physical-chemical properties (Table 5), only MTBE had concentrations above 5 ppb on the edge of the plume, while DCPD and 8-DHDCPD have significant concentrations just near the source.

Because of their hydrophobicity, DCPD (log $K_{ow}$ 2.9) and derivatives are expected not to move far from the polluted area, thus marking the spill origin and remaining undegraded for years. The extremely low odor threshold of this compound (100 ppt)[2] makes the contaminated water (free of other organic contents) unacceptable for drinking purposes, although it is moderately toxic to fish, algae, and a variety of other aquatic species.

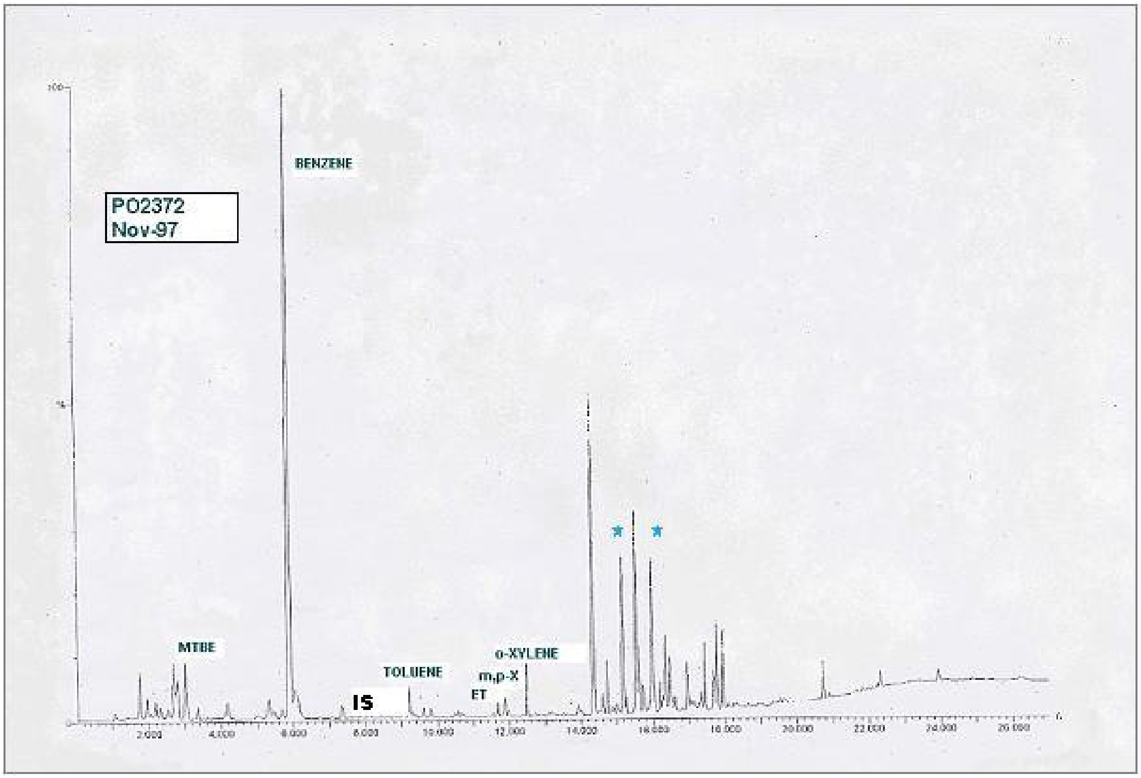

**FIGURE 2.** Chromatogram of well PO2372 taken in November 1997. Peaks assigned to DCPDs compounds are star marked. (IS = Internal standard).

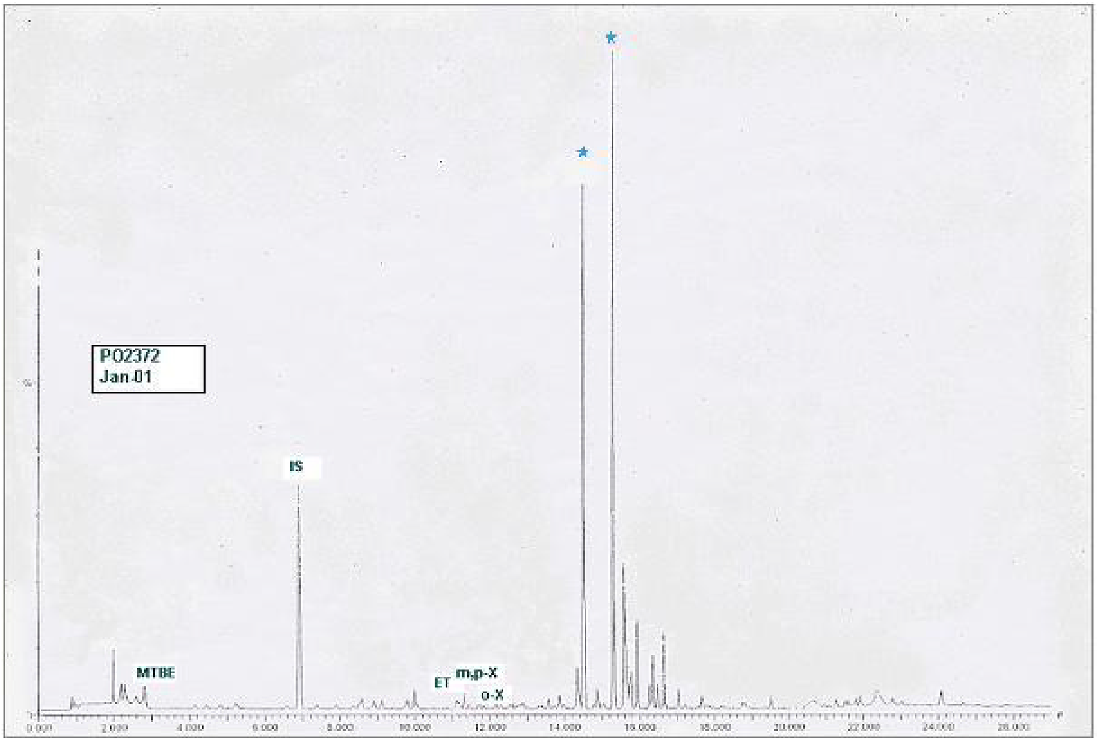

**FIGURE 3.** Chromatogram of well PO2372 taken in January 2001. Peaks assigned to DCPDs compounds are star marked. (IS = Internal standard).

**TABLE 4**

**Relationship Between Distances from the Spill Focus and Contaminant Analysis Profiles**

| march-01 | ppb | | | | | | | | |
|---|---|---|---|---|---|---|---|---|---|
| | | MTBE | BENZENE | TOLUENE | ETHYLBENZENE | *m+p*-XYLENE | *o*-XYLENE | DCPD | 8-DHDCPD | DCPD / 8-DHDCPD |
| 150 m | po2372 | <6 | 1.6 | <0.6 | 0.7 | 1.3 | <0.5 | 67.0 | 54.0 | 1.2 |
| 990 m | po2324 | 610 | <0.6 | <0.6 | <0.5 | <0.5 | <0.5 | 0.4 | <0.3 | _ |
| 1380 m | po2325 | 65.4 | <0.6 | <0.6 | <0.5 | <0.5 | <0.5 | 0.4 | 0.4 | 1.0 |
| 2400 m | po2327 | <6 | <0.6 | <0.6 | <0.5 | <0.5 | <0.5 | <0.3 | <0.3 | _ |

| oct-01 | ppb | | | | | | | | | |
|---|---|---|---|---|---|---|---|---|---|---|
| | | MTBE | BENZENE | TOLUENE | ETHYLBENZENE | *m+p*-XYLENE | *o*-XYLENE | DCPD | 8-DHDCPD | DCPD / 8-DHDCPD |
| 48 m | po9027 | 103 | 18.4 | 21.8 | <0.5 | 25.9 | 73.5 | 8.1 | 2.1 | 3.9 |
| 80 m | po9030 | 132 | 10.7 | 9.9 | 5.8 | 6.6 | 11.5 | 4.2 | 2.1 | 2.0 |
| 780 m | po4029 | 6.8 | <0.6 | <0.6 | <0.5 | <0.5 | <0.5 | <0.3 | <0.3 | _ |
| 990 m | po9555 | 6.7 | <0.6 | <0.6 | <0.5 | <0.5 | <0.5 | <0.3 | <0.3 | _ |

Top table corresponds to oil refinery storage tanks (La Pobla de Mafumet, March 2001); bottom table corresponds to a petrol service station spill (Sant Celoni, October 2001).

**TABLE 5**

**Physical-Chemical Properties**

| *Property* | *MTBE* | *Benzene* | *Toluene* | *Ethylbenzene* | *m+p-Xylene* | *o-Xylene* | *DCPD* |
|---|---|---|---|---|---|---|---|
| Molecular weight (g/mole) | 88.15 | 78.11 | 92.13 | 106.16 | 106.16 | 106.16 | 132.2 |
| Density, g/cm$^3$ | 0.744 | 0.88 | 0.867 | 0.867 | 0.884-0.8611 | 0.88 | 0.977 |
| Boiling temperature, ºC | 53.6 | 80.1 | 110.6 | 136.3 | 139.3-137.5 | 144.4 | 172.8 |
| Water solubility mg/l | 50000 | 1780 | 534.8 | 161 | 146-156 | 175 | - |
| Vapor pressure at 25ºC, mm Hg | 245 | 95.2 | 28.4 | 9.5 | 8.3-8.7 | 6.6 | 3.9* |
| Log $K_{ow}$ | 1.2 | 2.13 | 2.73 | 3.15 | 3.2-3.15 | 3.12 | 2.9** |

Values were reported from ref. [4].
*Calculated from log$p$ = 3.6172-2056.49/$T$, $p$ (Mpa) and T (K) [5].
**Ref. [2]

## CONCLUSION

Results obtained in the groundwater contamination episodes monitored near the source area show a rapid decay of BTEX contents (decreasing quickly to levels <0.5 ppb) that are in sharp contrast with the persistence of DCPD and 8-DHDCPD. Otherwise, MTBE had a slow decrease according to its known high solubility and recalcitrant behavior[9].

On the basis of such analytical profiles found in proximity to the contamination source, we propose to include DCPD and 8-DHDCPD as tracers of gasoline pollution when concentrations of BTEX and MTBE become clearly lowered. Purge-and-trap, followed by MS detection, was a sensitive method to monitor these compounds with a lower detection limit than MTBE; this one, a very soluble compound, had a poorer recovery in purge step.

Levels of DCPD and 8-DHDCPD found in all gasolines studied indicate that they are minor constituents. Their relative concentrations are similar in all formulations (2:1) except for 95-unleaded gasoline, which shows an inverse proportion (1:3). However, it is difficult to use the proportion of these compounds observed in real samples as gasoline-type markers. Further studies about their solubility, degradation, and behavior on soil and water matrices are still needed.

## ACKNOWLEDGEMENTS

This paper was presented at the CSIC/ESF Workshop Analysis, Toxicity and Biodegradation of Organic Pollutants in Groundwater from Contaminated Land, Landfills, and Sediments, Barcelona, Spain, 8–10 November, 2001. The authors are indebted to F. Ventura (AGBAR) for a generous supply of DCPD standard.
